# Association of prevalent vaginal microbiome of mother with occurrence of type I diabetes in child

**DOI:** 10.1038/s41598-018-37467-w

**Published:** 2019-01-30

**Authors:** Mysore V. Tejesvi, Ritva Nissi, Karita Saravesi, Anna Maria Pirttilä, Annamari Markkola, Anne Talvensaari-Mattila, Anna Liisa Ruotsalainen

**Affiliations:** 1 Department of Ecology and Genetics, PO Box 3000, University of Oulu, FI-90014 Oulu, Finland; 2 Department of Obstetrics and Gynecology, PO Box 5000, Oulu University Hospital, FI-90014 Oulu, Finland; 3 Chain Antimicrobials Ltd, Teknologiantie 2, FI-90590 Oulu, Finland

## Abstract

Type I diabetes (T1D) is a rapidly increasing autoimmune disease especially in the Western countries and poses a serious global health problem. Incidence of T1D cannot be fully explained by genetic background, and environmental factors have been assumed to play a role. Environmental conditions and composition of human microbiome have been found to correlate with the incidence of T1D. We asked whether mothers’ prevalent vaginal microbiome could correlate with the incidence of T1D in child. To test this hypothesis, we collected samples of vaginal microbiomes from eight mothers that had at least one child with T1D (child age maximum of 11 years at the time of sampling), born with a vaginal delivery. Eight control mothers had child/children with vaginal delivery and no diabetic child/children. The microbiomes were studied by using 16S rRNA Ion Torrent high throughput sequencing. We found that composition of total and *Lactobacillus* microbiome was altered, and saw an indication that diversity of vaginal microbiomes of the mothers with a diabetic child could be higher. Based on these pilot observations, we strongly encourage a larger population study to verify whether mother vaginal microbiome diversity and composition are linked to the prevalence of T1D in children.

## Introduction

Type I diabetes (T1D) is one of the most common pediatric chronic diseases^1^. The incidence of T1D has been increasing drastically especially in the population of the Western countries during the past decades^2–4^ and this increase has been most dramatic among the children of 0–4 years of age^2,4^. Globally, there is a high geographical variation in the incidence of T1D; the highest increase rates being reported in Europe^1^. Finland and Sardinia have the highest incidence of T1D, annually about 60 and 40 cases per 100 000 people, respectively^3^. Although very recent reports from Nordic countries indicate that the accelerating increase of T1D may be levelling off^5–7^, the incidence of T1D is still quite high in these countries^4,6^. The role of enterovirus infections preceding T1D has especially gained attention^8^, and a vaccination has been developed being in a testing phase at present^9^.

The genetic determination behind T1D is polygenic with at least 40 loci to date known to affect^10^. However, all genetically susceptible individuals do not get T1D as e.g. in Finland 20% of the population bears the susceptible HLA-mediated genes but only 1–2% will get T1D^11^. As all genetically susceptible individuals do not get T1D, the environmental factors therefore should play a role^4,12–14^. Several factors such as diet^15^, hygienic conditions^16–18^, vitamin D level^19^, enterovirus infections^8^ and gut microbiome^20,21^ have been found to correlate with autoimmunity and incidence of T1D. In particular, much attention has been paid to the alterations in gut microbiome as a potential factor in the predisposition of T1D^4^.

Human microbiome is primarily determined at the birth^22^ but changes in its composition also occur later in life^23–25^. The mode of delivery affects the microbiome of the neonate, i.e., children born via cesarean section have different microbial profiles than vaginally delivered ones^26–28^. Skin and vaginal bacterial communities are known to be dominated by different phyla^26,29–32^. Children born by cesarean section have been reported to have a 20% higher risk to get T1D^33^.

Healthy vaginal microbiome of a single individual, has been found to be inhabited by at least 30 bacterial groups, dominated by *Lactobacilli;* also including pathogenic bacterial groups in low numbers^34–37^. Communities may be highly individual^36,37^. The vaginal microbiome is considered to be relatively stable and resilient during the reproductive age until the menarche^36,37^, but temporal variation related to menstrual cycle, sexual activity and original bacterial community composition has been described^37–39^.

As the vaginal microbiome of the mother (i) may correlate with the microbiome of vaginally delivered neonates^22^, (ii) the mode of delivery is correlated with the child microbiome^26,27^ and (iii) because the child gut microbiome has been found to be altered in the progression of T1D^4^, we asked whether there would be systematic differences in the prevalent vaginal microbial communities between mothers with and without a T1D child/children. To investigate this hypothesis, we studied vaginal microbiomes from a pilot group of mothers in Northern Finland, Oulu area, by using 16S rRNA Ion Torrent sequencing. Specific attention was paid to *Lactobacillus* community.

## Results and Discussion

The total number of sequence reads was 251 431, and the average number per sample was 7857 ± 807.1 SD. For the subsequent analyses, the sequences were rarified into the lowest number of reads per sample (4341 reads). The reads were clustered into 185 operational taxonomic units (OTUs), representing seven identified phyla and 73 identified genera.

**Figure 1:**
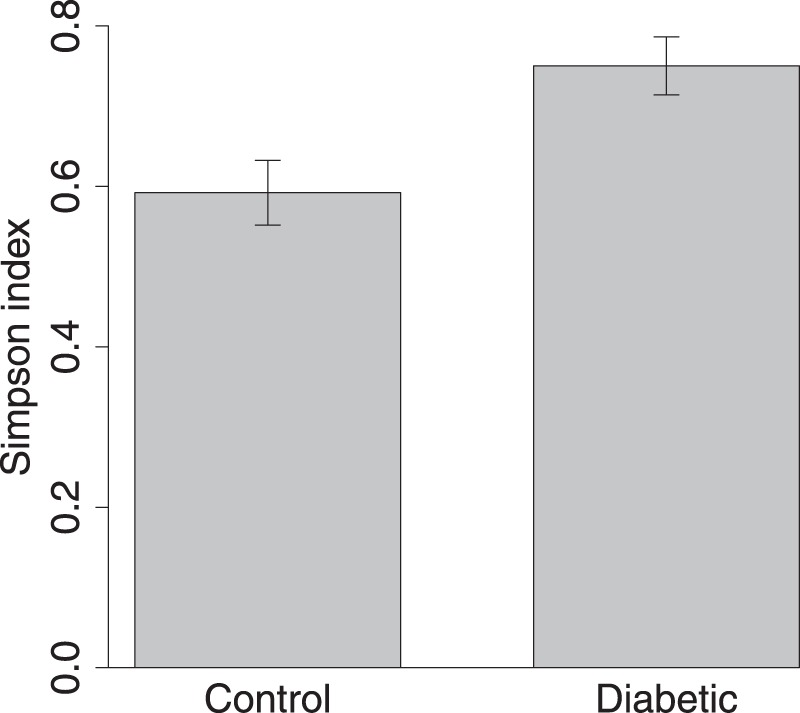
Vaginal microbiome Simpson’s diversity index (±SE). Control = in mothers without T1D child and Diabetic = in mothers with one or more child/children with T1D. F_(1,14)_ = 4.36, P = 0.055. N = 8.

**Figure 2:**
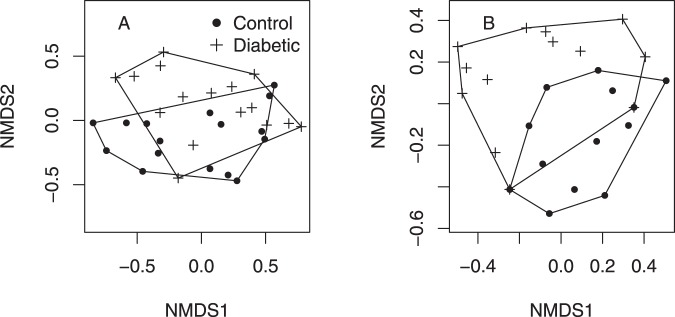
NMDS ordination of the microbiomes of control and diabetic samples based on presence/absence data. (**A**) All OTUs, PERMANOVA F_(1,30__)_ = 2.02, P = 0.049. (**B**) *Lactobacillus*-OTUs. PERMANOVA F_(1,30__)_ = 10.0, P < 0.001. N = 8 (16 microbiomes in the figure are based on swab type duplicates. Swab type was used as a random factor in the analysis).

**Figure 3:**
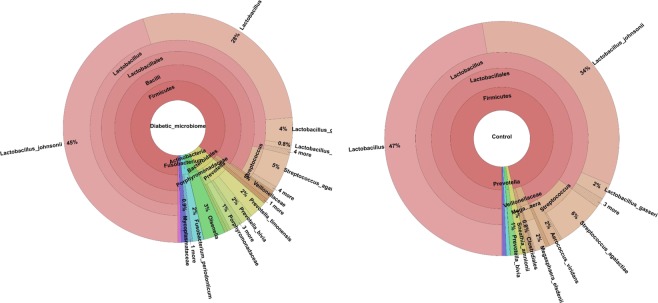
Microbiomes of mothers of diabetic and control groups visualized at different taxonomic levels. N = 8.

There was no difference in the total relative abundance of OTUs (Supplementary Information 1, 2 and 3), but there was an indication that the microbiome diversity may have been increased in the group of T1D mothers suggested by marginally significant Simpson diversity index (Fig. 1, F_(1,14)_ = 4.36 , P = 0.055). Chao1 and Shannon indices did not suggest differences between groups (Supplementary Information 2). The composition of microbiomes was altered (presence/absence data, PERMANOVA F_(1,30)_ = 2.02, P = 0.049, Figs 2A and 3). *Lactobacilli* were the most common OTUs in both studied groups (Supplementary Information 4), and the composition of *Lactobacillus* community was found to be altered (presence-absence data, PERMANOVA F_(1,30__)_ = 10.0, P < 0.001, Figs 2B and 3) between groups.

To our knowledge, this is the first comparison of vaginal microbiomes between mothers with a diabetic child versus mothers without diabetic child. Although the sample size is low (N = 8), we were able to find an indication that the prevalent vaginal microbiome of mothers with a T1D child may have higher OTU diversity than the vaginal microbiome of the control mothers and we found that the composition of microbiomes was different. Potential changes in diversity of T1D mother vaginal microbiomes were linked with alteration in the community of the dominant bacterial genus *Lactobacillus*^37^. Different *Lactobacillus* community, and decreased dominance of *Lactobacillus* in the vaginal bacterial flora may be linked to the more diverse microbial community in T1D mothers. In the gut microbiota, especially *L. johnsonii* abundance has been found to correlate negatively with the incidence of diabetes in a rat model^40,41^. De Goffau *et al*. reported increased microbial diversity in the gut of diabetic children (over 2.9 years) compared to non-diabetic children^42^, although it is known that in the late progression phase of T1D in humans, the gut microbiome becomes more monotonous^4^. The amount of *Lactobacilli* in the vagina is related to production of lactic acid and vaginal pH^37,39,43,44^.

The vaginal microbiome is considered being relatively stable and resilient during the reproductive age until the menarche^37^. However, temporal variation related to menstrual cycle, sexual activity and gestation have been found^37,39,44^. Therefore, factors such as mother age, age at birth of child/children, stage of menstrual cycle and information about the sexual activity should be controlled in the future studies. In contrast to earlier studies (e.g. Cho & Blaser)^22^, Drell *et al*. recently found that vaginal microbiome of the mother and the child microbiome (e.g. child gut microbiome) do not correlate with each other although they share same bacterial taxa^31^. There may thus also be other than microbiome-related mechanisms between increased risk of the child to get T1D than mother vaginal microbiome.

In addition, characteristic bacterial groups in the microbiomes of the mothers with a T1D child/children were Actinobacteria, Fusobacteria and *Prevotella*. *Prevotella* has been found common in the vaginal microbiome in women who have other than *Lactobacillus*-dominated microbiome^36^. Although other than *Lactobacillus*-dominated vaginal microbiomes are less common, they are considered as normal, *i.e*. healthy vaginal microbiomes^44^.

Our results, based on a small pilot study, suggest that vaginal microbiome of T1D mothers may differ from the mothers without a T1D child several years after delivery. They also provide material to develop a hypothesis that mother’s vaginal microbiome plays a role in the incidence of a T1D breakout in children and this may be related to increased microbial diversity in the vagina. More detailed research on the potential connection between the mother vaginal microbiome and the child T1D is therefore of major importance. We also suggest that vaginal seeding of neonates born by cesarean section^45^ should be applied with caution until we know whether a vaginal microbiome could pose a health risk related to T1D or other more common disease^46^. We emphasize that our results should be treated as indicative, and they need to be verified by a more extensive population study; especially with a more thorough background information collected from the study units (mothers), the age of child/children, and antibiotic treatments controlled.

## Methods

The material for this study was collected in the OYS hospital June 16 2014 - June 15 2015. Institutional committee of Pohjois-Pohjanmaan sairaanhoitopiirin kuntayhtymä approved the study and the patient consent (Statement of the regional Ethics committee February 18, 2012). All research was performed in accordance with relevant regulations, and informed consent was obtained from all participants. The material for the study was collected from 8 mothers with at least one T1D child by vaginal delivery (child age less than 11 years at the time of sampling and the child being diagnosed with diabetes at the age of 0–4 y old) and from 8 control mothers with at least one vaginal delivery and no diagnosed diabetic child/children. The age of the mothers was set to be between 22–40 years at the time of sampling. The samples were collected during in Oulu area, Northern Finland. No other background information on the mothers was collected.

As the type of the swab could affect the quality of the DNA, the samples were collected by using both Dacron (EnviroTrans) and cotton swabs (Constix) in a normal protocol of vaginal surface sampling. After sampling, the tip of the swab was immediately placed into a sterile Eppendorf tube and preserved at −80 °C.

### DNA extraction and amplification of bacterial rRNA genes

DNA was extracted from the dacron and cotton swabs using the Qiagen DNeasy Blood and Tissue kit (Qiagen, USA) according to manufacturer’s protocol and stored at −20 °C until used. The DNA was quantified using a Nanodrop spectrophotometer. Bacterial primers F519 (5-CAGCMGCCGCGGTAATWC-3) and R926 (5-CCGTCAATTCCTTTRAGTTT-3) were used to amplify the V4-V5 region of 16S small-subunit ribosomal gene. The F519 primer contained an Ion Torrent pyrosequencing adapter sequence A (Lifescience Technologies, USA), a 9-bp unique barcode sequence and one nucleotide linker, while the R926 primer contained an Ion Torrent adapter trP1 sequence. Triplicate PCR reactions were performed, each containing 1x Phusion GC buffer, 0.4 µM of the forward and reverse primers, 200 µM dNTPs, 0.5 U of Phusion enzyme (Finnzymes, Finland) and 10 ng of genomic community DNA as the template, together with molecular-grade water in a total reaction volume of 20 µl. The cycling conditions were 30 cycles at 98 °C for 10 s, 64 °C for 30 sec and 72 °C for 20 sec after an initial denaturation at 98 °C for 3 min. The triplicate pooled PCR amplified reactions were purified using the AMPure XP PCR clean-up kit (Agencourt Bioscience, CA, USA) and the DNA concentration was measured on a Bioanalyzer DNA chip (Agilent Technologies, CA, USA). Individual samples were pooled in an equivalent amount, size selected using BluePippin automated electrophoresis system (Sage Science, MA, USA) on 1.5% agarose gel, twice purified with AMPure XP kit, final DNA concentration was measured using Bioanalyzer DNA chip and sequenced on a 316 v2 chip using Ion Torrent 400 bp chemistry (Life Technologies, USA) using to 15 pM sample concentration.

### Bioinformatics analysis

The hypervariable regions V4-V5 of the 16S rRNA gene were sequenced using Ion Torrent to characterize the microbiomes of the vaginal samples. The Ion Torrent sequences were processed and analyzed with QIIME using state-of-the-art procedures^47^. Briefly, the sequences were binned according to sample-specific barcodes using the QIIME split_libraries.py tool, after which the barcode and primer sequences were trimmed and filtered for quality using the default parameters. Chimeric sequences were removed with the Usearch quality-filtering tool in QIIME using the rRNA16S.gold.fasta reference database. The final dataset consisted of 251 431 reads from 4 × 8 (32) samples after filtering out low-quality and chimeric readings, with a median of 7,612 reads per sample. The sequences were clustered into operational taxonomic units (OTUs) employing a similarity threshold of 97% in an RDP Naive Bayesian Classifier. The OTUs represent taxonomic units based on differences between sequences of bacterial DNA data. The total number of OTUs serves as an estimate of the total number of bacterial species in the sample but is not an exact equivalent of the number of different microbiological species. The phylogenetic trees were constructed from NAST-trimmed aligned sequences in FastTree2^48^. The OTU table was constructed using the Biom formatted table in QIIME v1.9 and all the samples were rarefied to 4300 sequences prior the OTU-based analysis, as it was the lowest number of readings observed in the community. Taxonomy was assigned using gut-specific microbiome HITdb^49^. We have deposited the raw Ion Torrent data in NCBI-SRA with the accession number SRP132770. Chao1, Shannon and Simpson indices were used to estimate the diversity of the microbiome. Data is available from the corresponding author on request.

### Statistical analysis

Before analyses, the sequences were rarified to the lowest number of sequences in a sample in QIIME. Because there were no differences between cotton and dacron swabs (Supplementary Information 2), these data were later combined in the analysis. Statistical analysis was conducted using a linear mixed effects model with the total OTU relative abundance, Chao1, Simpson and Shannon diversity indices as the dependent variables, and the mother group (control or with a T1D child) as an explanatory factor. The swab type was used as a random factor in the analyses and mother was used as an additional random factor (by nesting swab within mother) for OTU relative abundance and diversity indices. We carried out non-metric multidimensional scaling (NMDS) and the statistical test by PERMANOVA (functions metaMDS and adonis is package vegan 2.3.5^50^) analysis for the bacterial communities, both for relative abundance and presence/absence data. The fit of the statistical models was confirmed by using diagnostic plots^51^ and suitable transformation/s were carried out for the dependent variable when appropriate. The analyses were conducted in R-environment, v. 3.1^52^.

## Supplementary information

Supplementary information
