# Supplementary material for: Association of prevalent vaginal microbiome of mother with occurrence of type I diabetes in child
Source: Sci Rep. 2019 Jan 30;9:959. doi: 10.1038/s41598-018-37467-w (PMC6353987; doi:10.1038/s41598-018-37467-w)
Supplement: Supplementary file 1 — Supplementary information [file 41598_2018_37467_MOESM1_ESM.pdf]

Supplementary information

Association of prevalent vaginal microbiome of mother with occurrence of type I diabetes in child

Mysore V Tejesvi, Ritva Nissi, Karita Saravesi, Anna Maria Pirttilä, Annamari Markkola, Anne Talvensaari-Mattila, Anna Liisa Ruotsalainen

Supplementary information 1.

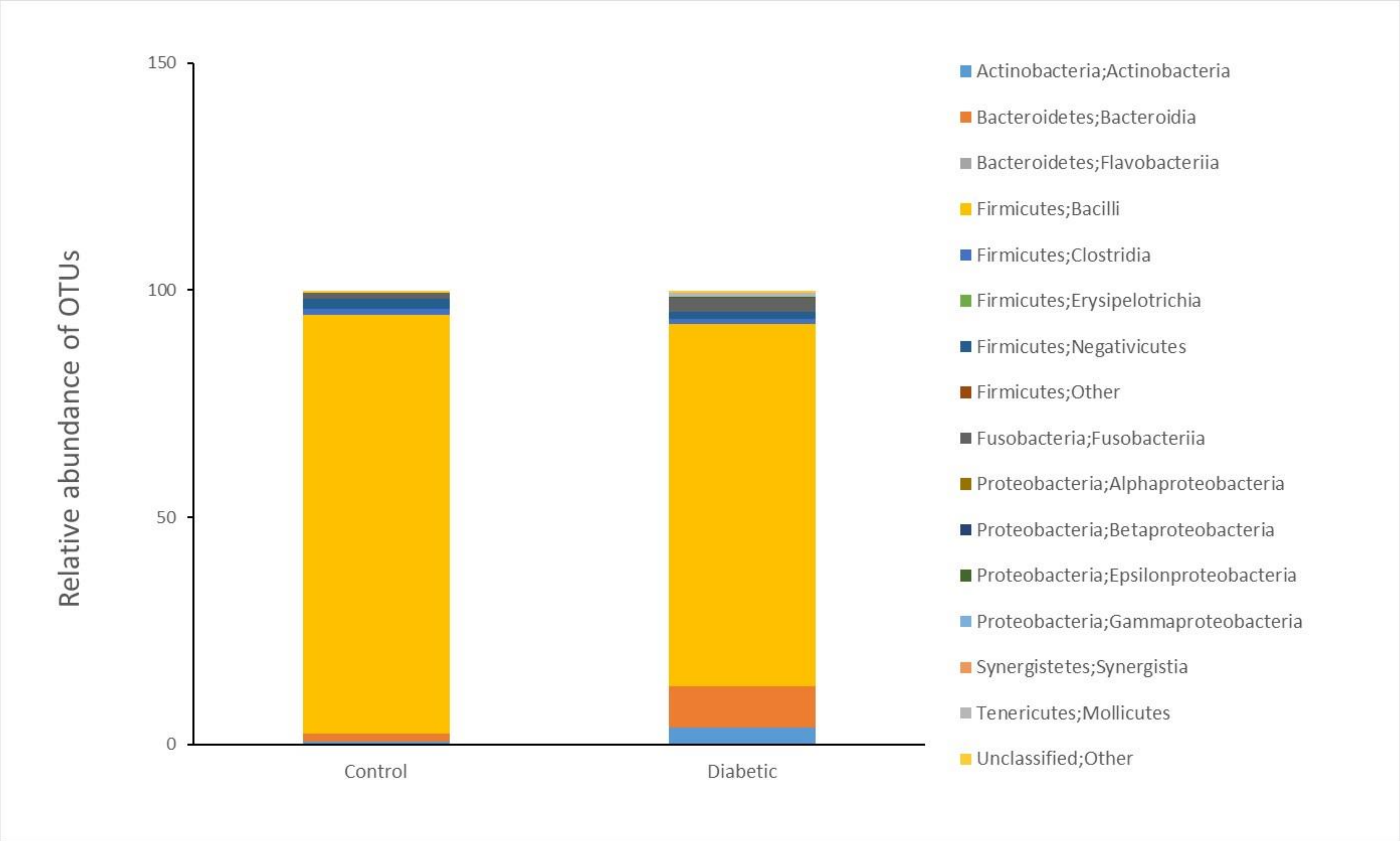

Supplementary information 2.

R scripts, outputs and diagnostic plots of analysis of variance (ANOVA) and linear mixed effects (LME) models.

1. Analysis of the impact of swab type on Chao1, Shannon and Simpson index and OTU relative abundance by using 1-way analysis of variance (ANOVA). Explanatory factor: swab type (levels: cotton and Dacron). Testing carried out for control and diabetic groups separately.
2. Testing the impact of mother group (= diab, levels: control = no diabetic child/children, diabetic = 1 or more T1D child/children).

1. Impact of swab:

Impact of swab type within the control group:

```
> model<-aov(chao~swab)

> summary(model)

            Df Sum Sq Mean Sq F value Pr(>F)

swab         1   8066    8066   0.181  0.677

Residuals   14 622179   44441
```

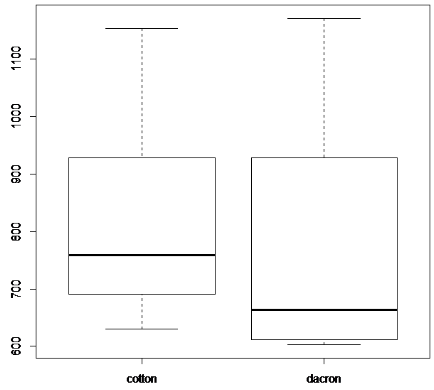


```
> model<-aov(simpson~swab)

> summary(model)
```

```
          Df Sum Sq  Mean Sq F value Pr(>F)
swab        1 0.0007 0.000711   0.026  0.875
Residuals   14 0.3901 0.027863
```

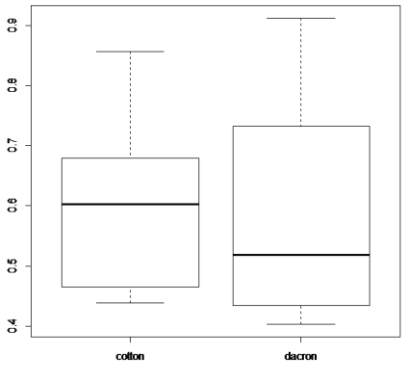


```
> model<-aov(shannon~swab)
> summary(model)
          Df Sum Sq Mean Sq F value Pr(>F)
swab        1  0.027  0.0275   0.026  0.873
Residuals   14 14.522  1.0373
```

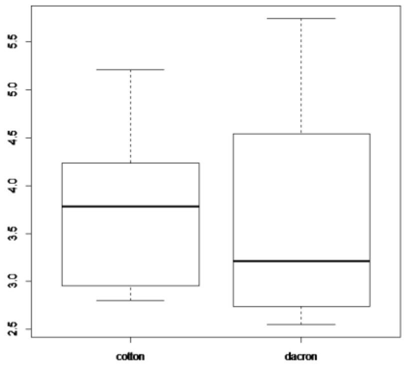


```
> model<-aov(otus~swab)
> summary(model)
          Df Sum Sq Mean Sq F value Pr(>F)
swab        1    420     420   0.045  0.835
Residuals   14 130976    9355
```

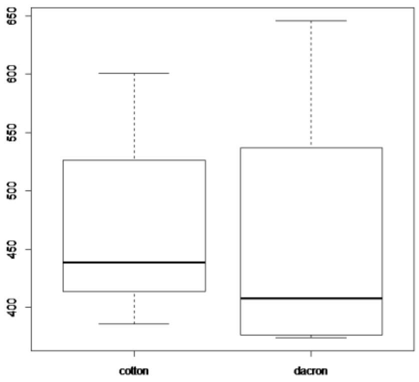

Impact of swab type within the diabetic -group:

Chao mary(model)

	Df	Sum Sq	Mean Sq	F value	Pr(>F)
swab	1	773	773	0.008	0.93
Residuals	14	1370532	97895		

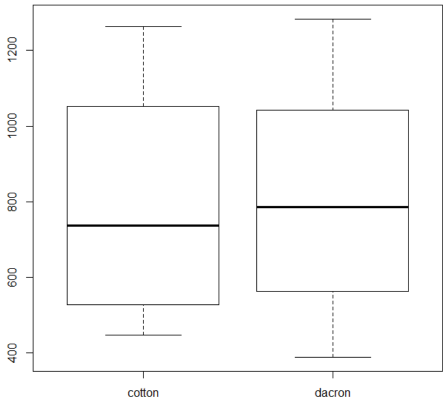

> model<-aov(simpson~swab)

> summary(model)

	Df	Sum Sq	Mean Sq	F value	Pr(>F)
swab	1	0.01512	0.01512	0.708	0.414
Residuals	14	0.29889	0.02135		

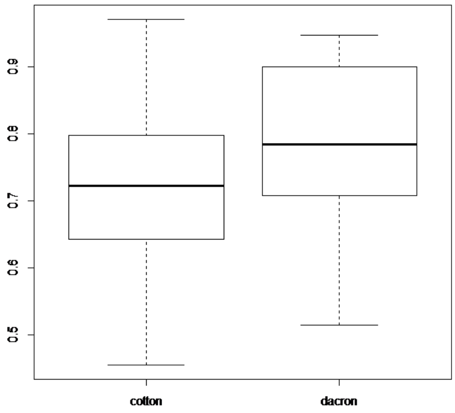


```
> model<-aov(shannon~swab)
> summary(model)
          Df Sum Sq Mean Sq F value Pr(>F)
swab       1  0.903  0.9026   0.579  0.459
Residuals 14 21.806  1.5576
```

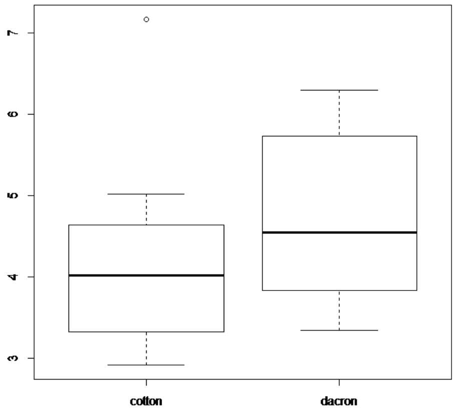


```
> model<-aov(otus~swab)
> summary(model)
          Df Sum Sq Mean Sq F value Pr(>F)
swab       1    743     743   0.035  0.855
Residuals 14 298727   21338
```

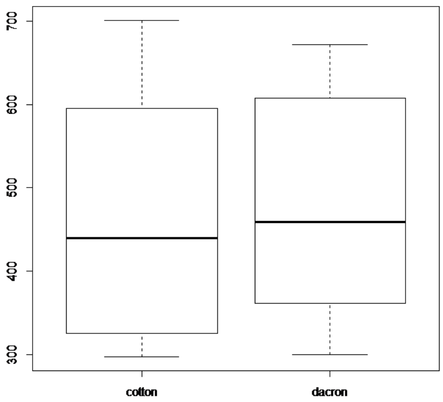

2. LME analysis with swab type nested within mother as random factors:

chao1:

```
> model<-lme(chao1~diab, random=~1|mother/swab)
> summary(model)
Linear mixed-effects model fit by REML
 Data: NULL
      AIC      BIC    logLik
  402.0949 409.1009 -196.0475

Random effects:
 Formula: ~1 | mother
        (Intercept)
StdDev:    257.4674

 Formula: ~1 | swab %in% mother
        (Intercept) Residual
StdDev:    56.79128   40.285

Fixed effects: chao1 ~ diab
```

```
              Value Std.Error DF  t-value p-value
(Intercept) 795.0355  92.67789 16 8.578480  0.0000
diaby         5.2664 131.06633 14 0.040181  0.9685
 Correlation:
      (Intr)
diaby -0.707

Standardized Within-Group Residuals:
        Min          Q1         Med          Q3         Max
-0.86810772 -0.29023812 -0.04126476  0.28423593  0.82173538

Number of Observations: 32
Number of Groups:
          mother swab %in% mother
              16              32
> anova(model, test="F")
            numDF denDF   F-value p-value
(Intercept)     1    16 148.15720  <.0001
diab            1    14   0.00161  0.9685
```

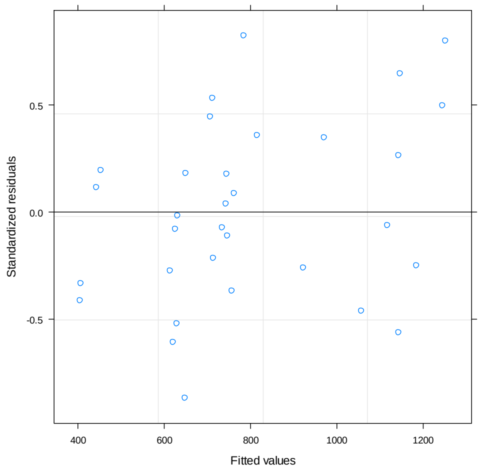

Shannon:

```
> model<-lme(log(shannon)~diab, random=~1|mother/swab)
> summary(model)
Linear mixed-effects model fit by REML
 Data: NULL
      AIC      BIC   logLik
  4.983506 11.98949 2.508247

Random effects:
 Formula: ~1 | mother
        (Intercept)
StdDev:   0.2358537

 Formula: ~1 | swab %in% mother
        (Intercept)   Residual
StdDev:   0.1091228 0.05912714

Fixed effects: log(shannon) ~ diab
               Value  Std.Error DF   t-value p-value
(Intercept) 1.2770332 0.08897251 16 14.353122  0.0000
diaby       0.1928538 0.12582613 14  1.532701  0.1476
 Correlation:
      (Intr)
diaby -0.707

Standardized Within-Group Residuals:
         Min          Q1          Med           Q3          Max
-0.840098596 -0.177552409 -0.004972501  0.142179216  0.822314774

Number of Observations: 32
```

Number of Groups:

mother swab %in% mother

16 32

> anova(model, test="F")

	numDF	denDF	F-value	p-value
(Intercept)	1	16	476.5961	<.0001
diab	1	14	2.3492	0.1476

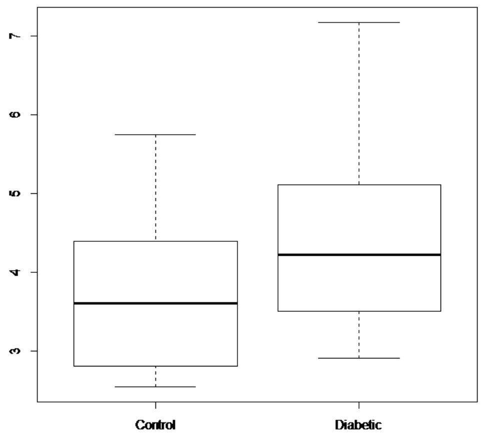

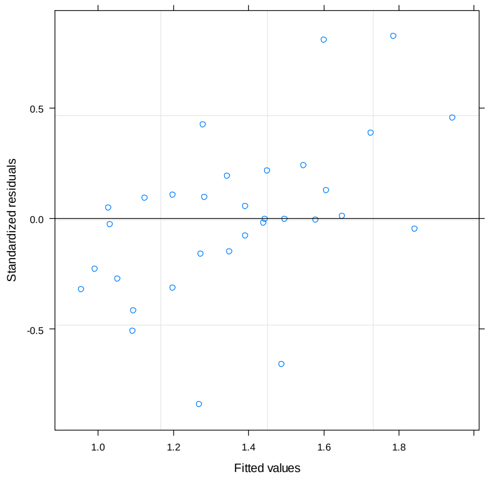

Simpson:

> model<-lme(simpson~diab, random=~1|mother/swab)

```
> summary(model)
Linear mixed-effects model fit by REML
 Data: NULL
       AIC       BIC   logLik
  -30.84982 -23.84383 20.42491

Random effects:
 Formula: ~1 | mother
       (Intercept)
StdDev:   0.1445313

 Formula: ~1 | swab %in% mother
       (Intercept)   Residual
StdDev:  0.05461761 0.03184253

Fixed effects: simpson ~ diab
              Value Std.Error DF   t-value p-value
(Intercept) 0.5921033 0.0534881 16 11.069813  0.0000
diaby       0.1580268 0.0756436 14  2.089096  0.0554
 Correlation:
     (Intr)
diaby -0.707

Standardized Within-Group Residuals:
        Min          Q1         Med          Q3         Max
-0.878460163 -0.205815669  0.006344226  0.164438159  0.898140447

Number of Observations: 32
Number of Groups:
         mother swab %in% mother
             16               32
```

```
> anova(model, test="F")
            numDF denDF   F-value p-value
(Intercept)     1    16 314.85578  <.0001
diab            1    14   4.36432  0.0554
```

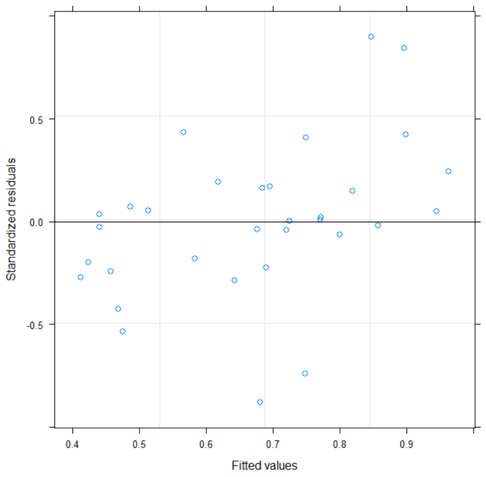

OTUs:

```
> model<-lme(observed_otus~diab, random=~1|mother/swab)
> summary(model)
Linear mixed-effects model fit by REML
 Data: NULL
       AIC      BIC    logLik
  351.3529 358.3589 -170.6765

Random effects:
 Formula: ~1 | mother
        (Intercept)
StdDev:    120.6659

 Formula: ~1 | swab %in% mother
        (Intercept) Residual
```

```
StdDev:    22.15855 16.78072

Fixed effects: observed_otus ~ diab
              Value Std.Error DF  t-value p-value
(Intercept) 463.0000  43.22408 16 10.71162   0.000
diaby         8.6875  61.12808 14  0.14212   0.889
 Correlation:
      (Intr)
diaby -0.707

Standardized Within-Group Residuals:
        Min          Q1         Med          Q3         Max
-0.85536882 -0.34326499 -0.06487676  0.31265093  1.01254874

Number of Observations: 32
Number of Groups:
          mother swab %in% mother
              16               32
> anova(model, test="F")
            numDF denDF  F-value p-value
(Intercept)     1    16 233.8037  <.0001
diab            1    14   0.0202   0.889
> plot(model)
```

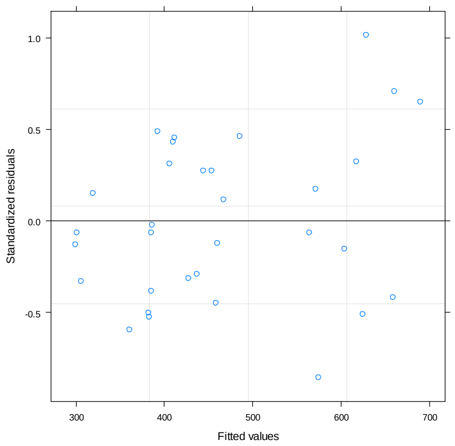
Standardized residuals
1.0
0.5
0.0
-0.5
300
400
500
600
700
Fitted values

Supplementary information 3.

R scripts and outputs of NMDS ordination and PERMANOVA (function *adonis* in R-program) analyses *based on the relative abundance of OTUs*. A: All OTUs. B: Lactobacillus OTUs. Degrees of freedom, F and p values are in bold.

A. <u>NMDS ordination of the microbiomes of control and diabetic samples. N = 8 (16 microbiomes based on swab type duplicates).</u>

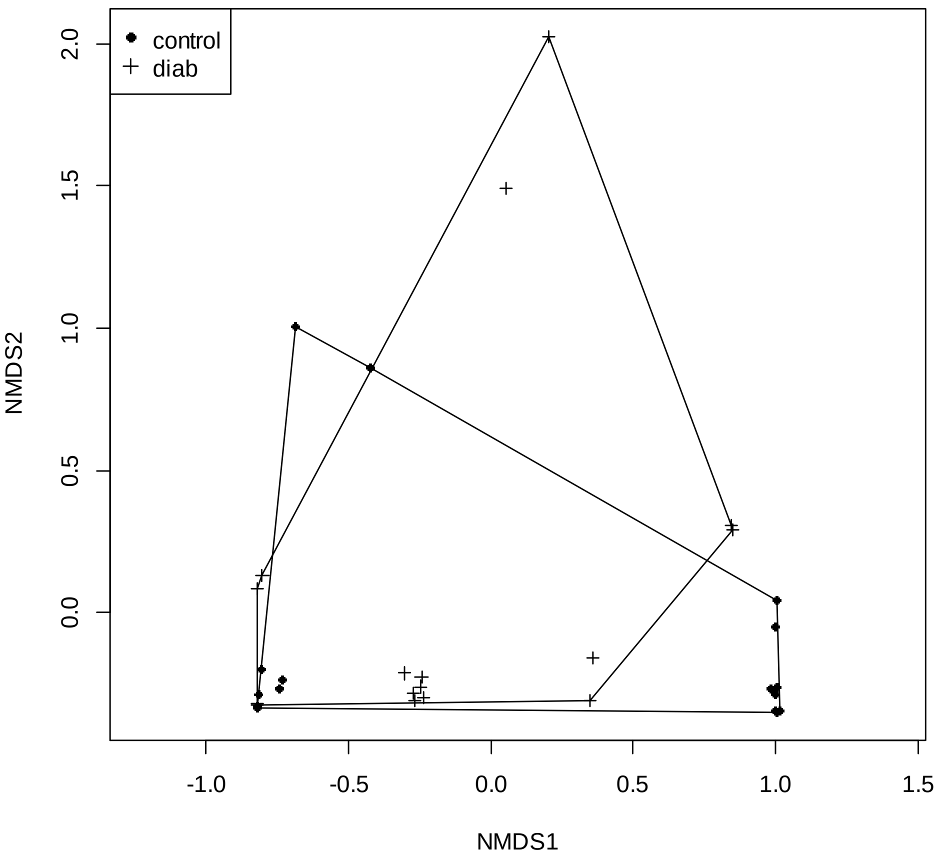

> attach(aineisto)

> diss<-vegdist(aineisto[,4:187])

> model<-adonis(diss~Treatment, strata=Swab, data=aineisto, permutations=9999)

> model

Call:

adonis(formula = diss ~ Treatment, data = aineisto, permutations = 9999,   strata = Swab)

Blocks: strata

Permutation: free

Number of permutations: 9999

Terms added sequentially (first to last)

	Df	SumsOfSqs	MeanSqs	F.Model	R2	Pr(>F)
Treatment	**1**	0.2739	0.27386	**1.2735**	0.04072	**0.2615**
Residuals	**30**	6.4513	0.21504		0.95928	
Total	31	6.7251			1.00000	

B. PERMANOVA and NMDS ordination of *Lactobacillus* OTUs based on their relative abundances

> diss <- vegdist(aineisto[,4:17])

> mod <- adonis(diss ~ Treatment, strata=Swab, data=aineisto, permutations=9999)

> mod

Call:

adonis(formula = diss ~ Treatment, data = aineisto, permutations = 9999,   strata = Swab)

Blocks: strata

Permutation: free

Number of permutations: 9999

Terms added sequentially (first to last)

	Df	SumsOfSqs	MeanSqs	F.Model	R2	Pr(>F)
Treatment	**1**	0.2489	0.24894	**1.3046**	0.04167	**0.2592**
Residuals	**30**	5.7247	0.19082		0.95833	

Total	31	5.9737	1.00000

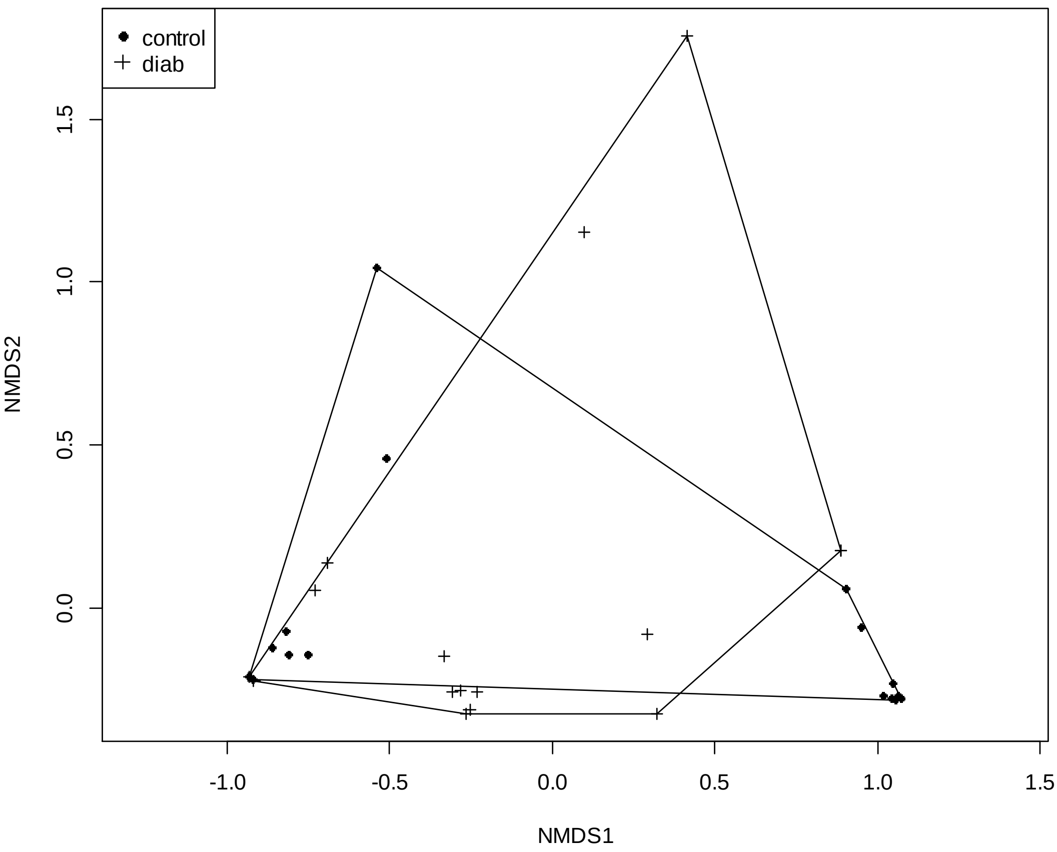

Supplementary information 4.

Twenty most abundant OTUs in the vaginal microbiomes of mothers who have not delivered a child/children with type 1 diabetes (Control) and mothers who have at least one child with type 1 diabetes (Diabetic).

Control	%	SD	Diabetic	%	SD
Firmicutes;Bacilli;Lactobacillales;Lactobacillaceae;Lactobacillus;Other	0.468	0.379	Firmicutes;Bacilli;Lactobacillales;Lactobacillaceae;Lactobacillus;OTU199|NN=Lactobacillus_johnsonii_HE573915|D=94.9	0.416	0.288
Firmicutes;Bacilli;Lactobacillales;Lactobacillaceae;Lactobacillus;OTU199|NN=Lactobacillus_johnsonii_HE573915|D=94.9	0.322	0.373	Firmicutes;Bacilli;Lactobacillales;Lactobacillaceae;Lactobacillus;Other	0.281	0.185
Firmicutes;Bacilli;Lactobacillales;Streptococcaceae;Streptococcus;Streptococcus_agalactiae	0.064	0.147	Firmicutes;Bacilli;Lactobacillales;Streptococcaceae;Streptococcus;Streptococcus_agalactiae	0.048	0.128
Actinobacteria;Actinobacteria;Coriobacteriales;Coriobacteriaceae;Olsenella;Other	0.022	0.022	Actinobacteria;Actinobacteria;Coriobacteriales;Coriobacteriaceae;Olsenella;Other	0.030	0.080
Firmicutes;Bacilli;Lactobacillales;Lactobacillaceae;Lactobacillus;OTU1294|NN=Lactobacillus_gasseri_JQ805634|D=96.7	0.017	0.045	Firmicutes;Bacilli;Lactobacillales;Lactobacillaceae;Lactobacillus;Lactobacillus_gasseri	0.024	0.037
Bacteroidetes;Bacteroidia;Bacteroidales;Prevotellaceae;Prevotella;Prevotella_bivia	0.016	0.018	Bacteroidetes;Bacteroidia;Bacteroidales;Prevotellaceae;Prevotella;Prevotella_timonensis	0.023	0.061
Firmicutes;Bacilli;Lactobacillales;Lactobacillaceae;Lactobacillus;OTU1032|NN=Lactobacillus_johnsonii_HE573915|D=95.8	0.013	0.034	Firmicutes;Bacilli;Lactobacillales;Lactobacillaceae;Lactobacillus;Lactobacillus_johnsonii	0.020	0.026
Firmicutes;Bacilli;Lactobacillales;Lactobacillaceae;Lactobacillus;Lactobacillus_gasseri	0.012	0.033	Bacteroidetes;Bacteroidia;Bacteroidales;Prevotellaceae;Prevotella;Prevotella_bivia	0.017	0.049
Bacteroidetes;Bacteroidia;Bacteroidales;Prevotellaceae;Prevotella;Prevotella_timonensis	0.010	0.023	Fusobacteria;Fusobacteriia;Fusobacteriales;Fusobacteriaceae;Fusobacterium;OTU23|NN=Fusobacterium_periodonticum_AB910749|D=96.5	0.014	0.042
Firmicutes;Bacilli;Lactobacillales;Lactobacillaceae;Lactobacillus;Lactobacillus_johnsonii	0.005	0.005	Bacteroidetes;Bacteroidia;Bacteroidales;Porphyromonadaceae;Other;Other	0.012	0.040
Firmicutes;Bacilli;Lactobacillales;Aerococcaceae;Aerococcus;Aerococcus_viridans	0.005	0.014	Firmicutes;Bacilli;Lactobacillales;Lactobacillaceae;Lactobacillus;OTU1294|NN=Lactobacillus_gasseri_JQ805634|D=96.7	0.011	0.024
Fusobacteria;Fusobacteriia;Fusobacteriales;Fusobacteriaceae;Fusobacterium;OTU23|NN=Fusobacterium_periodonticum_AB910749|D=96.5	0.005	0.006	Firmicutes;Bacilli;Lactobacillales;Lactobacillaceae;Lactobacillus;OTU1032|NN=Lactobacillus_johnsonii_HE573915|D=95.8	0.009	0.011
Fusobacteria;Fusobacteriia;Fusobacteriales;Leptotrichiaceae;Sneathia;Sneathia_amnionii	0.005	0.005	Firmicutes;Bacilli;Lactobacillales;Lactobacillaceae;Lactobacillus;Lactobacillus_oris	0.008	0.020

Bacteroidetes;Bacteroidia;Bacteroidales;Porphyromonadaceae;Other;Other	0.004	0.009	Fusobacteria;Fusobacteriia;Fusobacteriales;Fusobacteriaceae;Fusobacterium;Other	0.007	0.023
Firmicutes;Negativicutes;Selenomonadales;Veillonellaceae;Megasphaera;OTU301|NN=Megasphaera_elsdenii_DQ146765|D=93.8	0.004	0.004	Tenericutes;Mollicutes;Mycoplasmatales;Mycoplasmataceae;Mycoplasma;Mycoplasma_hominis	0.006	0.018
Unclassified;Other;Other;Other;Other;Other	0.003	0.009	Firmicutes;Negativicutes;Selenomonadales;Veillonellaceae;Dialister;OTU1084|NN=Dialister_succinatiphilus_AB370249|D=93.2	0.006	0.019
Firmicutes;Bacilli;Lactobacillales;Lactobacillaceae;Lactobacillus;Lactobacillus_oris	0.003	0.008	Unclassified;Other;Other;Other;Other;Other	0.005	0.004
Firmicutes;Negativicutes;Selenomonadales;Veillonellaceae;Dialister;OTU1084|NN=Dialister_succinatiphilus_AB370249|D=93.2	0.003	0.005	Firmicutes;Bacilli;Lactobacillales;Streptococcaceae;Streptococcus;Streptococcus_anginosus	0.004	0.010
Fusobacteria;Fusobacteriia;Fusobacteriales;Fusobacteriaceae;Fusobacterium;Other	0.002	0.006	Bacteroidetes;Bacteroidia;Bacteroidales;Other;Other;Other	0.003	0.009
**Sum**	**0.982**	**1.143**	**Sum**	**0.944**	**1.073**
